# Supplementary material for: Analysis of De Novo Mutations in Sporadic Cardiomyopathies Emphasizes Their Clinical Relevance and Points to Novel Candidate Genes
Source: J Clin Med. 2020 Jan 29;9(2):370. doi: 10.3390/jcm9020370 (PMC7073782; doi:10.3390/jcm9020370)
Supplement: Supplementary file 1 [file jcm-09-00370-s001.pdf]

Figure S1. The pedigrees of families with *de novo* variants in known cardiomyopathies genes and corresponding Sanger chromatograms. Family FD02 - p.Trp976Leu/c.2927G>T in *TTN*, family FD03 - p.Glu290Lys/c.868G>A in the *DSP*, family FD04 - p.Glu1548Gln/c.4642G>C in *SCN5A*, family FD06 - p.Glu94Val/c.281A>T in the *TNNC1*, family FD09 - p.Ile201Thr/c.602T>C in the *MYH7* (*de novo,* lower) and p.Lys205Arg/c.614A>G in *TPM1* (upper), family FD10 - p.Thr40Met/c.119C>T in *CRYAB*, family FR1 - p.Gly768Arg/c.2302G>A in the *MYH7* (*de novo,* lower) and p.Pro1066Arg/c.3197C>G in the *MYBPC3* (upper), family FH1 - p.Arg453Cys/c.1357C>T in the *MYH7*. Squares represent males and circles represent females. An arrowhead denotes the proband. Solid symbols denote affected status, open symbols with an asterisk denote not-affected individuals examined by Sanger sequencing. The presence or absence of a mutation in examined individuals is indicated by a + or − symbol respectively.

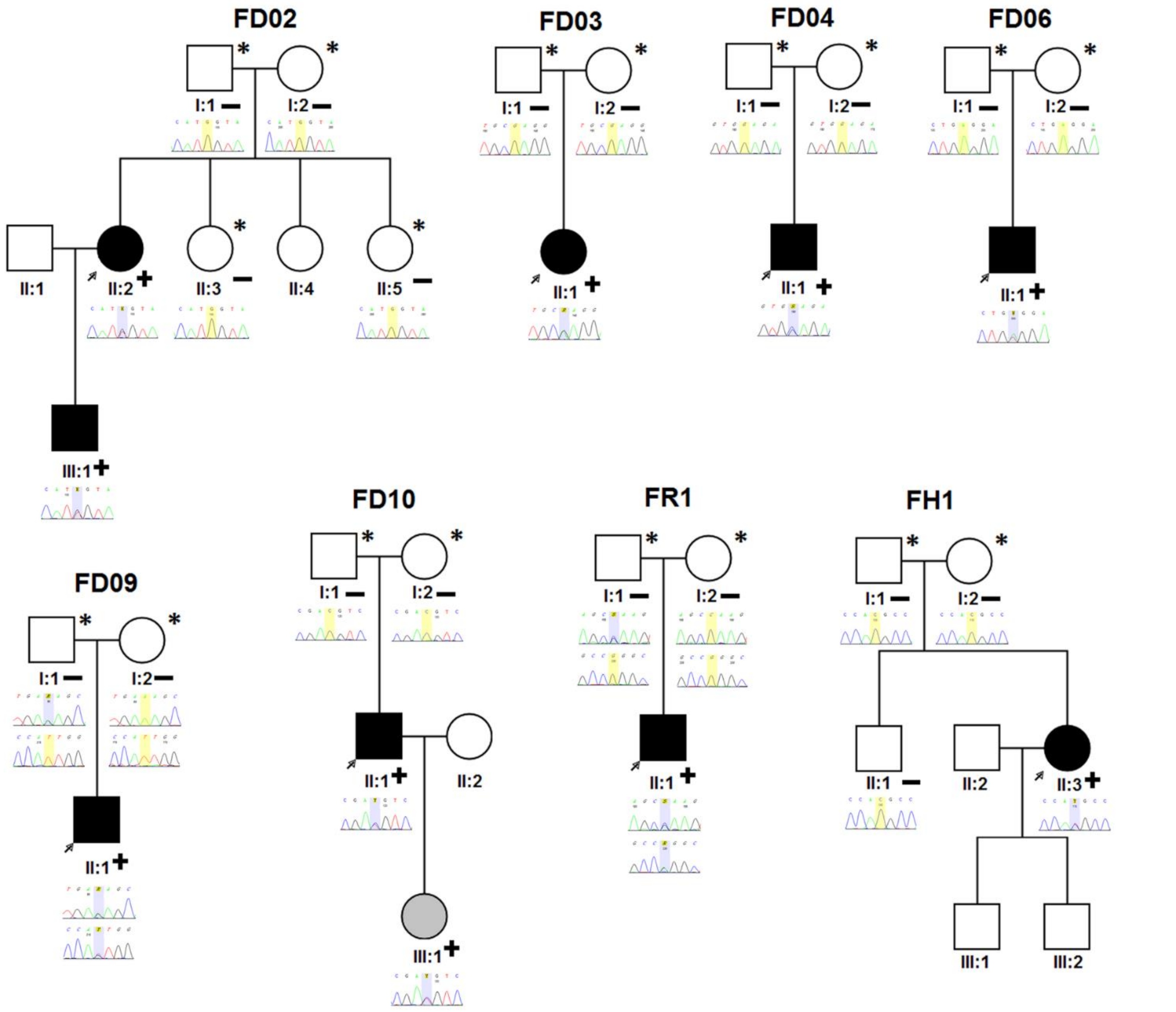

Table S1. Technical parameters of WES in trios

	% of target covered min. 20x	mean coverage	probes covered <10x /all probes (%)	WES enrichment used
FD01 proband	92.2	128.92	11107/229827 (4.8)	SureSelectXT Human All Exon v5 (Agilent)
FD01 father	95.5	101.41	4150/214414 (1.9)	SureSelectXT Human All Exon v5 (Agilent)
FD01 mother	95.6	95.76	3870/214414 (1.8)	SureSelectXT Human All Exon v5 (Agilent)
FD05 proband	84.3	45.93	4039/225534 (1.8)	SeqCap EZ MedExome (Roche)
FD05 father	89.9	54.47	2792/225534 (1.2)	SeqCap EZ MedExome (Roche)
FD05 mother	91.7	61.13	2299/225534 (1.0)	SeqCap EZ MedExome (Roche)
FD07 proband	93.1	67.39	3307/225534 (1.5)	SeqCap EZ MedExome (Roche)
FD07 father	90.3	57.14	2802/225534 (1.2)	SeqCap EZ MedExome (Roche)
FD07 mother	91.2	59.07	2545/225534 (1.1)	SeqCap EZ MedExome (Roche)
FD08 proband	93.3	68.95	2762/225534 (1.2)	SeqCap EZ MedExome (Roche)
FD08 father	93.6	65.28	2140/225534 (0.9)	SeqCap EZ MedExome (Roche)
FD08 mother	92.8	63.28	2033/225534 (0.9)	SeqCap EZ MedExome (Roche)

Table S2. The detailed clinical characteristics of probands

Family	Sex	Type of cardiomyopathy	Age at onset of symptoms/ screening	Age at diagnosis	Symptoms at onset	NYHA at diagnosis	LVEF (%)	LVDD	Other important echocardiographic data	CMR study	ECG	Arrhythmia	PM/ICD/CRT-D/RF ablation	Outcome	Additional abnormalities
Probands with *de novo* mutation in known cardiomyopathies gene															
FD02	F	DCM	34	34	DOE	II	30	70	No	LVEDVI 186ml/m2, LVEF 33%, normal RV, TAPSE 24mm, LGE(+)	SR, LV hypertrophy, ST-T changes	Infrequent, single VE	No	Slow improvement of LVEF on ACEI +BB, at age 39y, LVEF 42%, LVEDD 61mm	NA
FD03	F	Arrhythmogenic DCM	26	28	Palpitations, DOE	II	40	54	No	LVEDVI 113ml/m2, LVEF 37%, normal RV,TAPSE 20 mm, LGE(+)	SR, ST-T changes	complex VE, >12000, >600 couplets, 17nsVT	At age 30y ICD-VR,	At age 33y LVEF 45%, LVEDD 49mm, normal RV	Myocarditis diagnosed in the past, co-existent autoimmune nodular thyroid disease
FD04	M	Arrhythmogenic, incipient DCM	15	15	Palpitations, DOE	II	48	60	No	LVEDVI 121ml/m2, LVEF 56%, RVEDVI 119ml/m2, RVEF 51%, LGE (-)	SR	PAFIcomplex VE, nsVT	RF ablation of VA at age 17y, and 22y and CTI due to PAFI at age 22 y	At age 23y, on BB +ACEI, LEF 46%, LVEDD 56mm,	NA
FD06	M	early onset DCM	13	16	Acute dyspnea and symptoms of cardiogenic shock	IV	15	75	mild/moderate MI, dilated hypokinetic RV, TAPSE 11mm	Not available	SR, biatrial enlargement, LVH, ST-T changes in lateral and inferior leads	NA	No	OHT at age 16y following 3 months on BiVAD	NA
FD09	M	DCM with LVNC	19	19	Palpitations, DOE	I	26	62	No	LVEDVI 137ml/m2, LVEF 33%, the ratio of NC/C layer 3.4:1, nondilated RV, TAPSE 21mm, LGE(-)	SR, LAD, LVH	Few single VE and SVE	No	At age 24y mild exercise intolerance, on ACEI+BB, LVEF 37%, LVEDD 59mm	NA
FD010	M	DCM with LVNC and cardiac conduction system disease	36	36	Palpitations, dyspnea at rest, transient sensory aphasia due to ischemic stroke in the course of PAF	III	25	80	Prominent trabeculations of the middle and basal segments of the infero-lateral wall	LVEDVI 355.7ml/m2, LVEF 12%, , Ratio of NC/C layer >3 in the apical anterior, inferior and lateral segments, in the mid-ventricular segments: anterior, antero-lateral, inferolateral>2-2.5, LGE (+)	SR, IVCD, paroxysmal III degree A-V block	PAF, single VE 48/24h	ICD-DR at age 37y, upgrade to CRT-D at age of 38y	Improved, at age 43y stable, on ACEI +BB+spiro+D with LVEF 46%, LVEDD 67mm	Thyreotoxicosis in the past, shortsightedness - 4.5D with astigmatism
FR1	M	clinically RCM, morphologically hypoplastic left ventricle, and hypoplastic entire aorta, hypertrophied right ventricle due to pulmonary hypertension	28	29	As	III	60	41	Pulmonary hypertension of 60mmHg	LVEDVI 41ml/m2, LVEF 80%, RVEDVI 46ml/m2, RVEF 55%, mild IM with regurgitant fraction of 14%, dilated inferior vena cava, hypoplastic aorta, aortic root 22mm, STJ 18mm, ascending aorta 21mm, descending aorta 18mm, iliac arteries of 6-7mm, LGE (+)	SR, both atria enlargement	Infrequent single VE and few SVT	No	At age 35y mild exercise tolerance, pulmonary artery hypertension	Congenital defect of the urinary system, cirrhotic right kidney

FH1	F	Nonobstructive HCM with prominent restrictive features	16	34	DOE, at age of 32y ankle edema following 2nd delivery	III	40	55	asymmetric septal reverse curve hypertrophy with maximal thickness of the middle segment of 16mm, dilated both atria, SAM without obstruction, mild MI, reduced LV function	LVEDVI 109ml/m2, LVEF 49%, myocardial septal hypertrophy ("reverse curve") of the IVS of max. 15mm, thinning of the myocardial wall of middle inferolateral basal segments;, dilated atria LA 38cm2, RA 26cm2 LGE(+), extensive intramural and subendocardial in mid-ventricular and basal inferolateral segments	SR, LVH, AV I degree , IVCD	Infrequent single VE, 8 short SVT	At age 35y ICD-VR	Progressive HF symptoms, OHT at age 35y	NA
Probands with variants in novel DCM candidate genes															
FD01	M	Arrhythmogenic DCM	31	31	Palpitations, DOE	III	20	84	Mild MI	Not performed	SR, IVCD, negative T waves in inferolateral leads.	VE 20605/24h, 453 couplets, 13 nsVT	At age 31y ICD-VR, RF ablation for VA at age 32y, first PAF at age 34y	At age 36y died after experiencing acute abdominal pain, possibly due to an embolic episode. Autopsy not performed	Obesity, goiter. Positive family history for hypertension and obesity
FD05	F	DCM	40	40	DOE	III	15	65	Moderate MI	Not performed	SR, diffuse ST-T changes	VE 462/24h, 3 couplets	ICD-VR at age 40y	At age 44y with persisting left ventricular dysfunction	Hyperlipidemia
FD07[1]	F	Arrhythmogenic DCM	2	2	Recurrent infections, tiredness, palpitations	II	30	67	Moderate MI	dilated LV 165ml/m^2, and decreased LVEF of 38%, normal RV, LGE(+)	SR, negative T waves in lateral leads	VE 3894/24h, 192 couplets, 2 nsVT (5)	A history of VA ablation at age 21y	At age 26y stable left ventricular dysfunction, persistent ventricular arrhythmia	No
FD08	F	Arrhythmogenic DCM	21	29	Palpitations	II	38	53	No	LVEDVI 139ml/m2, LVEF 34%, LGE (+), diffuse subepicardial areas in the lateral wall and in the IVS.	SR, low QRS voltage in limb leads, diffuse ST-T changes	VE 2798/24h, 20 couplets 2 nsVT	ICD-VR at age 31y	At age 31y, stable with ventricular arrhythmia and LV dysfunction.	polycystic ovary syndrome; a history of eosinophilic myocarditis, diagnosed in local hospital by CMR and blood eosinophilia following parasitic infestation at age 28y

[1] Results of clinical assessment obtained at the time of genetic inquest, at age of 24y

Legend: ACEI – angiotensin-converting-enzyme inhibitor; As – asymptomatic; AV – atrioventricular; BB – betablokers; BiVAD – biventricular assist device; CMR – cardiac magnetic resonance; CRT-D – cardiac resynchronization therapy defibrillator; D-diuretic; DCM – dilated cardiomyopathy; DOE – dyspnea on effort; ECG – electrocardiogram; F – female; HCM – hypertrophic cardiomyopathy; HF – heart failure; ICD – implantable cardioverter defibrillator; ICD-VR - single chamber implantable cardioverter-defibrillator; LA – left atrium; LBBB – left bundle branch block; LGE – late gadolinium enhancement; LV – left ventricle; LVAD – left ventricular assist device; LVDD – left ventricular diastolic dysfunction; LVEDD - left ventricular end-diastolic diameter; LVEDVI – left ventricular end-diastolic volume index; LVEF – left ventricular ejection fraction; LVH – left ventricular hypertrophy; LVNC – left ventricular non-compaction; M – male; MI – mitral insufficiency; nsVT – non-sustained ventricular tachycardia; NYHA – New York Heart Association; OHT – heart transplantation; PAF – paroxysmal atrial fibrillation; PAFl - paroxysmal atrial flutter; PM – pacemaker; RA – right atrium; RCM – restrictive cardiomyopathy; RF – radiofrequency; RV – right ventricle; RVEDVI – right ventricular end-diastolic volume index; RVEF – right ventricular ejection fraction; SAM – systolic anterior motion; spiro – spironol; SR – sinus rhythm; SVT – supraventricular tachycardia; TAPSE – tricuspid annular plane systolic excursion; VE – ventricular ectopics.

Table S3: Other rare (<0,00001 in gnomAD) variants identified in probands as inherited from a parent

Family	Gene	Genomic coordinates (GRCh38)	Origin
FD01	GPATCH3	1:026900016-G>A	mat
FD01	LRRC53	1:074480860-C>T	pat
FD01	OR6Y1	1:158547257-GTAGA>G	pat
FD01	BROX	1:222725555-G>A	pat
FD01	TTC7A	2:047023416-A>G	mat
FD01	LIPT1	2:099162249-C>T	pat
FD01	SGPP2	2:222558797-CTGGAGAT>C	mat
FD01	COL6A3	2:237368676-A>G	mat
FD01	GOLGB1	3:121668125-G>T	mat
FD01	PLCH1	3:155514845-T>C	pat
FD01	ACSL1	4:184765964-C>T	mat
FD01	DST	6:056618910-C>G	mat
FD01	ZNF425	7:149103913-A>G	mat
FD01	SSPO	7:149823418-C>T	mat
FD01	PRAG1	8:008339549-C>A	mat
FD01	ADAM28	8:024323866-A>C	pat
FD01	CSPP1	8:067095686-G>T	pat
FD01	UBAP2	9:033933489-C>G	pat
FD01	ERCC6L2	9:095954820-C>T	pat
FD01	NUTM2G	9:096931793-T>G	pat
FD01	URM1	9:128389678-T>C	mat
FD01	WDR34	9:128640897-C>A	mat
FD01	CACNB2	10:018518409-G>A	mat
FD01	TIMM23B	10:049952454-G>A	pat
FD01	UROS	10:125788921-G>C	mat
FD01	AHNAK	11:062521585-A>T	pat
FD01	EMSY	11:076472741-G>A	mat
FD01	SLCO1C1	12:020721861-A>G	pat
FD01	POLE	12:132668822-T>G	mat
FD01	NID2	14:052068825-G>A	pat
FD01	SNRPA1	15:101285000-A>C	mat
FD01	CAPN15	16:000548179-G>A	pat
FD01	LCMT1	16:025126116-A>T	mat
FD01	WDR59	16:074893774-G>T	pat
FD01	SUPT6H	17:028683631-G>A	mat
FD01	DSG1	18:031346194-G>C	pat
FD01	TLE6	19:002982167-A>G	pat
FD01	LSR	19:035267546-GACAACGGCTCC>G	pat
FD01	ZNF480	19:052322847-C>T	pat
FD01	SOGA1	20:036793376-G>A	mat
FD01	HNF4A	20:044429640-C>A	pat
FD01	ATP9A	20:051690740-C>T	pat
FD01	ABCG1	21:042282275-T>C	pat
FD01	CABIN1	22:024072488-G>T	mat
FD05	TMEM183A	1:203007502-C>G	mat
FD05	ANKRD36C	2:095855358-A>C	mat
FD05	TTN	2:178741541-A>C	mat
FD05	STK36	2:218685196-A>G	pat
FD05	TRANK1	3:036864432-T>C	mat
FD05	ECE2	3:184257613-G>A	pat
FD05	MMAA	4:145654144-G>A	mat
FD05	CUL9	6:043224299-G>A	mat
FD05	RIMS1	6:072182975-A>G	pat
FD05	STEAP1B	7:022438530-T>C	mat
FD05	ZNF623	8:143650921-A>G	pat
FD05	PCSK5	9:076328127-G>C	pat
FD05	GTF3C4	9:132688886-G>A	pat
FD05	PTEN	10:087957919-G>A	pat
FD05	API5	11:043328857-A>G	pat
FD05	FAM180B	11:047588408-G>C	mat
FD05	FCHSD2	11:072867932-A>G	pat
FD05	MAP6	11:075668020-G>C	pat
FD05	NCAPD3	11:134203169-G>A	mat
FD05	OR6C68	12:055492735-C>T	pat
FD05	KIF5A	12:057581890-G>A	mat
FD05	HSP90B1	12:103942576-T>C	pat
FD05	MCF2L	13:113082428-G>T	mat
FD05	PAPLN	14:073262677-G>A	mat
FD05	PDXDC1	16:015006563-G>T	pat
FD05	ZNF48	16:030398724-T>C	mat
FD05	LOXHD1	18:046566408-A>T	pat
FD05	KHSRP	19:006415656-C>G	pat
FD05	STRN4	19:046725368-A>T	mat
FD05	CHD6	20:041420612-T>G	mat
FD05	Z82190.2	22:031804873-G>A	pat
FD07	TMEM82	1:015747029-C>CGAT	mat
FD07	GPATCH3	1:026892496-C>T	mat
FD07	INSL5	1:066801076-T>C	pat
FD07	LRRIQ3	1:074041792-G>C	pat
FD07	TMOD4	1:151174751-A>G	pat
FD07	UFC1	1:161154019-C>T	pat
FD07	B3GALT2	1:193180616-C>A	mat

FD07	OBSCN	1:228283746-C>T	pat
FD07	KIF3C	2:025929355-G>C	pat
FD07	PIGF	2:046612254-T>A	mat
FD07	PNPT1	2:055693667-T>C	mat
FD07	TRIM43	2:095594395-CA>C	pat
FD07	MERTK	2:111982905-T>A	mat
FD07	TEX264	3:051699427-C>A	mat
FD07	MYH15	3:108416882-A>G	mat
FD07	GOLGB1	3:121677372-CTGATCT>C	mat
FD07	IFT122	3:129514504-A>C	pat
FD07	PLSCR5	3:146594036-A>G	pat
FD07	KLHL24	3:183663508-T>A	mat
FD07	TADA2B	4:007054692-A>G	mat
FD07	PCDHB12	5:141209931-G>A	pat
FD07	ZNF354C	5:179078833-TAGA>T	pat
FD07	FOXC1	6:001612070-C>G	mat
FD07	HIST1H2BI	6:026273195-A>G	pat
FD07	EYS	6:063726546-C>G	mat
FD07	IGF2R	6:160088036-A>G	mat
FD07	ZNF107	7:064708623-G>C	mat
FD07	IMMP2L	7:110663680-CAGG>C	mat
FD07	IFRD1	7:112472256-C>T	pat
FD07	RBM33	7:155706911-G>A	pat
FD07	VCPIP1	8:066666955-A>C	mat
FD07	ABCA1	9:104845497-G>A	pat
FD07	ARHGAP22	10:048451332-T>C	mat
FD07	ERCC6	10:049471094-T>A	pat
FD07	OR4C16	11:055572972-C>T	mat
FD07	SPTBN2	11:066705253-C>G	mat
FD07	ADAMTS8	11:130416189-A>C	mat
FD07	PPM1A	14:060285761-A>AT	mat
FD07	RYR3	15:033810591-A>G	mat
FD07	MYZAP	15:057661476-A>T	pat
FD07	KBTBD13	15:065077342-C>G	mat
FD07	GOT2	16:058734225-C>T	mat
FD07	CARD14	17:080195604-G>A	pat
FD07	GRIN3B	19:001004906-C>T	mat
FD07	HIPK4	19:040380850-TG>T	mat
FD07	ZNF865	19:055615536-G>A	pat
FD07	RAD21L1	20:001240356-G>GA	pat

FD08	PHF13	1:006620223-C>G	mat
FD08	SLC25A34	1:015736624-C>G	pat
FD08	RASGRP3	2:033527402-A>G	pat
FD08	DYNC2LI1	2:043810402-A>G	pat
FD08	LOC102724058	2:166015611-A>G	pat
FD08	EDEM1	3:005187998-G>T	mat
FD08	NELFA	4:002008025-G>C	pat
FD08	PSAPL1	4:007434073-C>G	mat
FD08	GALNTL6	4:172813642-G>A	mat
FD08	PGGT1B	5:115221967-C>G	mat
FD08	ALDH7A1	5:126575429-G>A	pat
FD08	P4HA2	5:132195013-C>T	mat
FD08	ATAT1	6:030640423-G>GT	pat
FD08	DPCR1	6:030950094-A>T	mat
FD08	IGF2R	6:160084133-G>A	mat
FD08	ABHD11	7:073738778-AG>A	pat
FD08	IRF5	7:128948730-G>C	mat
FD08	KMT2C	7:152154375-C>A	pat
FD08	RNF170	8:042850999-T>C	mat
FD08	SAXO1	9:018928590-G>A	pat
FD08	GRIN1	9:137157024-C>A	mat
FD08	SORCS3	10:105139442-G>C	pat
FD08	AHNAK	11:062524576-G>C	mat
FD08	TRAPPC4	11:119023374-C>CT	pat
FD08	ST14	11:130198332-C>T	mat
FD08	EIF4B	12:053018815-A>G	mat
FD08	ATP4B	13:113649403-C>G	mat
FD08	RASGRP1	15:038499071-C>A	pat
FD08	TMEM62	15:043184548-A>C	mat
FD08	ALPK3	15:084857422-C>T	mat
FD08	PRSS53	16:031085241-TG>T	pat
FD08	PLCG2	16:081919531-A>C	mat
FD08	TEKT1	17:006819259-CT>C	mat
FD08	SLC35B1	17:049706209-T>C	mat
FD08	ABCC3	17:050659238-A>G	mat
FD08	FASN	17:082087170-G>C	pat
FD08	ZNF585B	19:037190131-T>G	mat
FD08	ZNF585B	19:037190138-A>G	mat
FD08	PSMC4	19:039974413-G>A	pat
FD08	GALR3	22:037824793-G>C	pat

Table S4. The ages of parents of probands' with *de novo* mutations

Family	Age at proband's birth	
	mother	father
FD01	26	27
FD02	24	26
FD03	28	27
FD04	20	40
FD05	18	27
FD06	23	21
FD07	20	21
FD08	31	33
FD09	25	25
FD10	27	26
FR1	25	40
FH1	28	25
